# Accelerating Photocatalytic Hydrogen Production and Pollutant Degradation by Functionalizing g-C_3_N_4_ With SnO_2_

**DOI:** 10.3389/fchem.2019.00941

**Published:** 2020-02-18

**Authors:** Amir Zada, Muhammad Khan, Muhammad Nasimullah Qureshi, Shu-yuan Liu, Ruidan Wang

**Affiliations:** ^1^Institute of Catalysis for Energy and Environment, College of Chemistry and Chemical Engineering, Shenyang Normal University, Shenyang, China; ^2^Department of Chemistry, Abdul Wali Khan University Mardan, Mardan, Pakistan; ^3^School of Materials Science and Engineering, Northwestern Polytechnical University, Xi'an, China; ^4^Department of Chemistry, University of Swabi, Swabi, Pakistan; ^5^Department of Pharmacology, Shenyang Medical College, Shenyang, China; ^6^Key Laboratory for Photonic and Electronic Bandgap Materials, Ministry of Education, College of Physics and Electronic Engineering, Harbin Normal University, Harbin, China

**Keywords:** g-C_3_N_4_, SnO_2_, photocatalysis, hydrogen production, organic pollutant

## Abstract

Energy crises and environmental pollution are two serious threats to modern society. To overcome these problems, graphitic carbon nitride (g-C_3_N_4_) nanosheets were fabricated and functionalized with SnO_2_ nanoparticles to produce H_2_ from water splitting and degrade 2-chlorophenol under visible light irradiation. The fabricated samples showed enhanced photocatalytic activities for both H_2_ evolution and pollutant degradation as compared to bare g-C_3_N_4_ and SnO_2_. These enhanced photoactivities are attributed to the fast charge separation as the excited electrons transfer from g-C_3_N_4_ to the conduction band of SnO_2_. This enhanced charge separation has been confirmed by the photoluminescence spectra, steady state surface photovoltage spectroscopic measurement, and formed hydroxyl radicals. It is believed that this work will provide a feasible route to synthesize photocatalysts for improved energy production and environmental purification.

## Introduction

Exhaustion of hydrocarbon fuels and addition of toxic and hazardous materials from agricultural, medicinal, dyes, and cosmetic industries to the environment have resulted in increased pressure on the scientific community to address these problems adequately. A number of methods have been chalked out such as cracking of hydrocarbons and thermal splitting of water at elevated temperature to get H_2_ (future fuel). However, these methods require highly costly and controlled operational environment and huge labor under normal conditions. On the other hand, different pollutants removal technologies such as adsorption, coagulation, and electrochemical methods have their own shortcomings and did not receive much popularity in the purification of the environment (Zhao et al., 2015; Gautam et al., 2016; Li et al., 2016; Ali et al., 2018b; Wang et al., 2018; Ali S. et al., 2019). Therefore, modern techniques are urgently required to address energy and environmental issues properly with the least operational cost and time.

Photocatalysis has opened a brilliant chapter to realize the energy crises and environmental issues. The photocatalysts have shown their remarkable influence in the production of H_2_ from water, production of hydrocarbon fuels from CO_2_, and removal of pollutants from air and wastewater with minimum cost and least working labor (Singh et al., 2017; Li et al., 2018a,b; Qu et al., 2018; Ullah et al., 2019; Xu et al., 2019a). Although different photocatalysts such as TiO_2_, ZnO, and ZrO_2_ have been widely utilized, there are some typical problems such as activeness only under ultraviolet (UV) light and fast recombination of photogenerated charges (Raizada et al., 2017; Qi et al., 2018a,b, 2020a). Since visible light contributes a major portion to electromagnetic radiations, therefore, photocatalysts active under visible light are more effective and efficient. Graphitic carbon nitride, g-C_3_N_4_, is a promising polymeric photocatalyst with a band gap of 2.7 eV. Its conduction band (CB) and valence band (VB) have characteristic abilities to reduce water and oxidize organic pollutants, respectively (Qi et al., 2019a,b). Its metal-free nature is of particular importance as its release to the environment does not produce harmful threats to the aquatic animals and plants (Nie et al., 2018; Ran et al., 2018; Fu et al., 2019; Liu M. et al., 2019). However, low surface area and poor excited charge separation capability of this photocatalyst is still a marked question on its utilization for fuel production and organic oxidation (Dong et al., 2019; Liu Y. et al., 2019; Zhu et al., 2019c; Qi et al., 2020b). Therefore, these problems need to be tackled in future generation of semiconductor photocatalysis.

SnO_2_ is an excellent UV responsive metal oxide photocatalyst with a band gap of 3.5 eV. Its excellent stability and tunable optical properties make it a suitable candidate for photocatalysis, solar cells, and gas sensors. More interestingly, its CB is situated at a suitable position below the CB of g-C_3_N_4_ and thermodynamically acts as a sink to accept the excited electrons from g-C_3_N_4_ during photocatalysis (Jana and Mondal, 2014; Xu et al., 2018a; Qi et al., 2019c,d). Therefore, its heterojunctional combination with g-C_3_N_4_ will significantly improve excited charge separation for enhanced photocatalysis.

In this work, we coupled SnO_2_ nanoparticles with g-C_3_N_4_ to form different ratio composites and applied for the photocatalytic production of H_2_ and 2-chlorophenol (2-CP) degradation under visible light, keeping in view to excite only g-C_3_N_4_ and use SnO_2_ as excited charge acceptor. The optimized composite (6SnO_2_/g-C_3_N_4_) showed much improved photoactivities for H_2_ production and pollutant degradation compared to bare SnO_2_ and g-C_3_N_4_. These activities are solely attributed to the better charge separation in the composites.

## Experimental Part

### Preparation of g-C_3_N_4_

Polymeric g-C_3_N_4_ was prepared from dicyandiamide. A given amount of dicyandiamide was taken in crucible and heated at 550°C in a muffle furnace for 4 h at the rate of 2°C/min. After the completion of the heating duration, the cooled sample was grinded into fine powder and used for further study.

### Preparation of SnO_2_ Nanoparticles

Chloride salt of Sn(IV) was dissolved in water, and the solution was made basic with the help of NaOH solution under vigorous stirring. During addition of NaOH, the solution initially became milky and then became clear with the addition of more NaOH. Finally, when the pH reached about 12, the solution was taken in an autoclave and heated at 220°C for 10 h in oven. The obtained white powder was purified three times with distilled water, dried in the oven overnight, and then calcined at 450°C for 1 h at the rate of 5°C/min.

### Preparation of SnO_2_/g-C_3_N_4_ Composites

Composites containing different mass percent of SnO_2_ and g-C_3_N_4_ (SnO_2_/g-C_3_N_4_) were prepared by taking appropriate amounts of SnO_2_ and g-C_3_N_4_ in water–methanol mixture containing 1 ml of concentrated HNO_3_. Each mixture was vigorously stirred under heating at 80°C till the whole solvent was evaporated. After that, each mixture was dried in the oven overnight and then calcined at 450°C for 1 h. The as prepared composites were represented by XSnO_2_/g-C_3_N_4_ where “X” shows the percent amount (2, 4, 6, and 8%) of SnO_2_ in the given composite.

### Characterization Techniques

The prepared samples were passed through different characterization techniques to confirm the formation of heterojunctional composites. The X-ray diffraction (XRD) technique was used to determine the crystalline structure of the samples with the help of Bruker D8 Advance diffractometer using CuK_α_ radiation. The oxidation states of the constituent elements of the composites were examined by means of X-ray photoelectron spectroscopy (XPS) employing X-ray from mono-Al source with the help of a Kratos-Axis Ultra DLD apparatus. The obtained binding energies were calibrated with the binding energy of adventitious C-atom which is 284.55 eV. The microscopic structure was further revealed with the help of transmission electron microscope (TEM) and high-resolution TEM (HRTEM) operating at 200 kV. The optical properties were confirmed with the help of UV diffuse reflectance spectra, by taking BaSO_4_ as a reference, measured with a Shimadzu UV-2550 spectrophotometer. The photoluminescence (PL) spectrum of each sample was realized with the help of spectrofluorophotometer (Perkin-Elmer LS55) at a 390-nm excitation wavelength. The steady state surface photovoltage spectroscopic (SS-SPS) measurement of each sample was carried in a controlled atmosphere employing a homemade equipment possessing a lock-in amplifier (SR830) synchronized with a light chopper (SR540). Each sample was first thoroughly grinded and then kept between two indium tin oxide (ITO) glass electrodes in an atmosphere-controlled sealed container. Radiations from a 500-W Xe lamp (CHF XQ500W, Global Xe lamp power) were passed through a double-prism monochromator (SBP300) to get a monochromatic light.

### Evaluation of Photoactivity for Water Splitting

Water splitting photocatalysis was carried out with the help of an online H_2_ production unit (Perfectlight, Beijing, Lab Solar III). About 0.1 g photocatalyst was taken in a glass-made reaction cell, and 100-ml aqueous solution of methanol (20% V/V) was added. The apparatus was first deaerated with the help of a vacuum pump to remove any traces of O_2_ and CO_2_ dissolved in aqueous solution. The mixture was irradiated under visible light (wavelength > 420 nm) coming from a 300-W Xe lamp under vigorous stirring. The produced gases were analyzed after each hour with the help of a gas chromatograph (7,900, TCD, molecular sieve 5 Å, N_2_ carrier, Tec comp.).

### Evaluation of Photoactivity for Pollutant Degradation

The photoactivities were further studied by selecting 2-CP as a pollutant. About 0.2 g of powder sample was mixed with 50 ml of aqueous solution (25 mg/L) of 2-CP and exposed to a 150-W (GYZ220) Xe lamp under visible light (wavelength > 420 nm). Before being exposed to light, each sample was first kept in complete dark for 30 min to attain adsorption–desorption equilibrium. The concentration of the pollutant was checked after each hour with the help of a Shimadzu UV-2550 spectrometer.

### Evaluation of Hydroxyl Radicals (·OH)

About 0.05 g powder sample was mixed with 50 ml of aqueous solution of coumarin (0.001 M) and exposed to a 150-W (GYZ220) Xe lamp under visible light (wavelength > 420 nm). Before exposure to light, each sample was first kept in complete dark for 30 min to attain adsorption–desorption equilibrium. After each hour, the amount of formed 7-hydroxycoumarin was checked at 390-/460-nm excitation/emission wavelengths with the help of a spectrofluorophotometer (Perkin-Elmer LS55).

## Result and Discussion

### Structural Characterization

**Figure 1:**
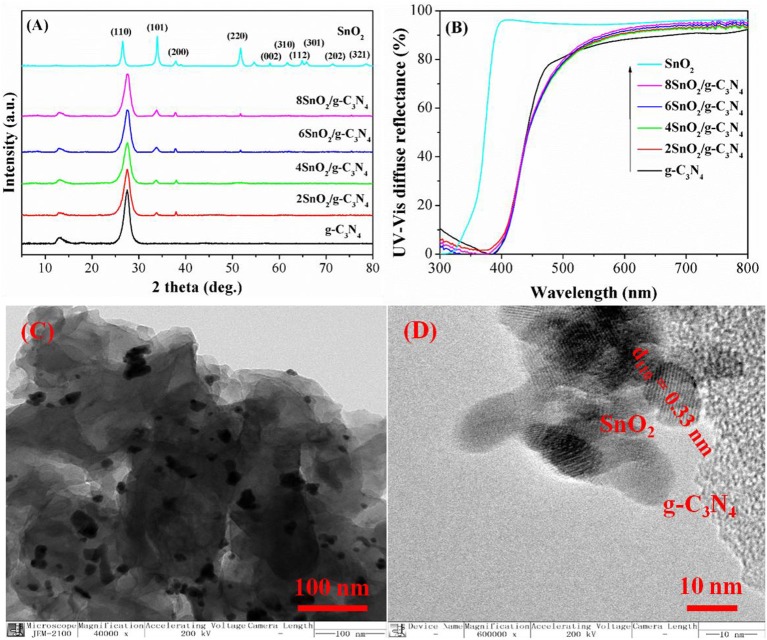
X-ray diffraction (XRD) pattern **(A)** and diffuse reflectance spectroscopy (DRS) **(B)** of g-C_3_N_4_, SnO_2_, and XSnO_2_/g-C_3_N_4_. Transmission electron microscope (TEM) image **(C)** and high-resolution TEM (HRTEM) images **(D)** of 6SnO_2_/g-C_3_N_4_.

The crystal structure study of the pure g-C_3_N_4_ shows two characteristic diffraction peaks at 13.04 and 27.31° as shown in Figure 1A. The former peak is due to the interplanner stacking of the aromatic rings in conjugation while the later peak is related to the interlayer structural units (Liu et al., 2017; Guan et al., 2018; Xu et al., 2018b, 2019b). Similarly, pure SnO_2_ shows diffraction peaks at 26.2, 33.8, 37.3, 51.2, 57.2, 61.1, 63.81, 64.77, 71.38, and 78.27°, which can be, respectively, indexed to (110), (101), (200), (220), (002), (310), (112), (301), (202), and (321) planes of tetragonal SnO_2_ nanoparticles (Mahjouri et al., 2020; Shokrzadeh et al., 2020). Interestingly, all the composite samples show the two characteristic peaks of g-C_3_N_4_ at 13.04 and 27.31° and SnO_2_ peaks at 33.8, 37.3, and 51.2°. However, the diffraction peak at 13.04° has been decreased progressively as the amount of SnO_2_ is increased, indicating that SnO_2_ nanoparticles are well packed in the nanosheets of g-C_3_N_4_.

The UV-vis diffused reflectance spectra of the samples are shown in Figure 1B. As can be seen, g-C_3_N_4_ and SnO_2_, respectively, show optical thresholds at 475 and 360 nm, corresponding to band gaps of 2.61 and 3.45 eV, respectively. Although the composite samples show the same optical thresholds at 475 and 360 nm, their light absorption has been slightly decreased as SnO_2_ is a wide-band-gap semiconductor and its coupling with g-C_3_N_4_ slightly decreases light absorption (Zhang et al., 2018; Zada et al., 2019a; Zhu et al., 2019a,b).

The TEM image of composite shows uniform distribution of small SnO_2_ nanoparticles of about 10-nm size on the surface of g-C_3_N_4_ as shown in Figure 1C. The HRTEM image shows the lattice fringes of 0.33-nm interplanar distance corresponding to the (110) plane of SnO_2_ (Figure 1D). This shows that both g-C_3_N_4_ and SnO_2_ are in close contact with each other to intensify the charge separation for better photoactivities.

**Figure 2:**
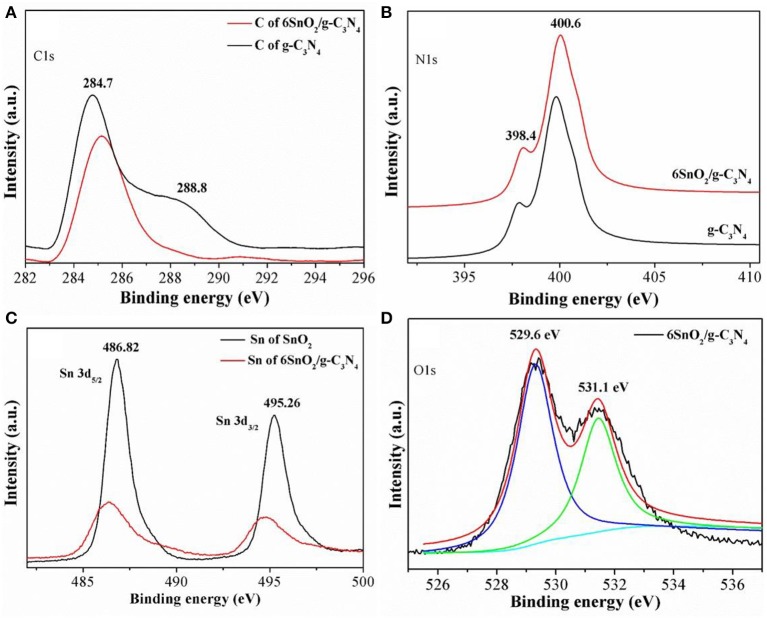
X-ray photoelectron spectra of C1s **(A)**, N1s **(B)**, Sn 3d **(C)**, and O1s **(D)** of g-C_3_N_4_ and SnO_2_.

The oxidation states of different elements in the samples were determined using XPS measurements, and the results are shown in Figure 2. The obtained binding energies were calibrated with the binding energy of the adventitious carbon atom with a binding energy value of 284.55 eV. It is obvious that C1s in pure g-C_3_N_4_ shows two XPS peaks at binding energies of 284.7 and 288.2 eV (Figure 2A). These peaks are attributed to the sp^2^ hybridized C-atoms, respectively, bonded with N-atom of the aromatic ring and NH_2_ group linking different aromatic rings. Similarly, the XPS binding energies of N1s in pure g-C_3_N_4_ are composed of two parts at 398.4 and 400.6 eV and are, respectively, contributed by sec. and ter. N-atoms (Figure 2B) (Raziq et al., 2016; Li Q. et al., 2019). The XPS peaks of Sn in pure SnO_2_ are deconvoluted into two parts at 486.82 and 495.26 eV, which are, respectively, contributed by Sn3d_5/2_ and Sn3d_3/2_ and show that Sn is present in +4 oxidation state in the nanocomposite (Figure 2C) (Li H. et al., 2019). When g-C_3_N_4_ nanosheets are coupled with SnO_2_ nanoparticles, the C1s and N1s peaks are slightly shifted toward the low-energy side while those of Sn are shifted toward the high-energy side. The binding energies of O1s in Figure 2D are contributed at 529.6 and 531.1 eV, which are, respectively, contributed by the lattice O-atom and adsorbed oxygen molecules. The redistribution of charge density in both components of the nanocomposite indicates that both g-C_3_N_4_ and SnO_2_ are present in close contact with each other for improved charge separation.

### Photocatalytic Activity

**Figure 3:**
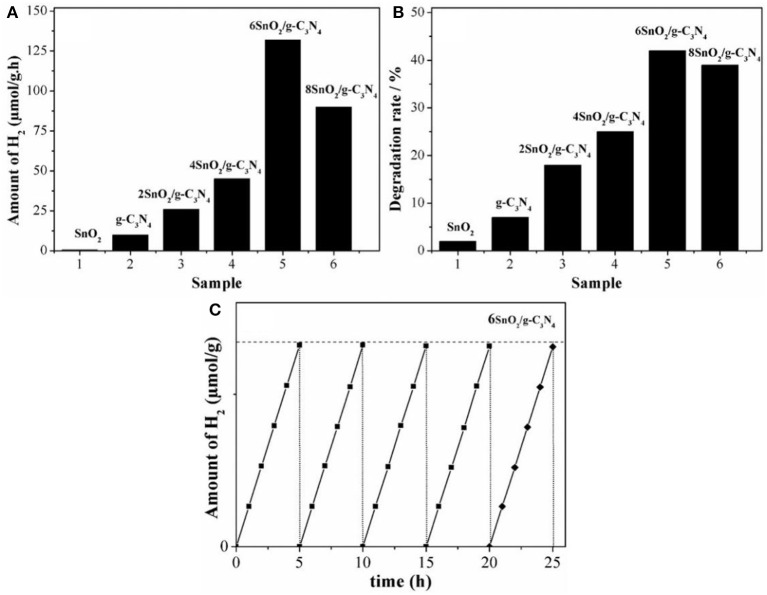
Photoactivities for H_2_ evolution **(A)**, 2-CP degradation **(B)** of pure g-C_3_N_4_, and XSnO_2_/g-C_3_N_4_ and stability test of 6SnO_2_/g-C_3_N_4_ **(C)**.

The photoactivities of composites were first evaluated by splitting water under visible light (wavelength > 420 nm) in the presence of methyl alcohol. As shown in the Figure 3A, the H_2_ production activity of pure SnO_2_ is negligible under visible light irradiation. However, pure g-C_3_N_4_ produces about 10 μmol of H_2_ in 1 h under the stipulated conditions. Interestingly, these H_2_ photoactivities are significantly enhanced when both g-C_3_N_4_ and SnO_2_ are coupled to form heterojunctional composites. Further, photoactivities are increased along with the increase in the amount of SnO_2_ nanoparticles and the highest activities are contributed by 6SnO_2_/g-C_3_N_4_ sample, which are 132 μmol/h. However, further increase in the amount of SnO_2_ decreases H_2_ production as SnO_2_ is a wide-band-gap semiconductor and it covers most surface of the g-C_3_N_4_ to prevent absorption of visible-light photons. These enhanced H_2_ activities are solely attributed to the improved charge separation in the composites by transferring excited electrons from g-C_3_N_4_ to SnO_2_ for the reduction of water. We further extended the photoactivities by measuring the degradation of 2-CP under visible-light (wavelength > 420 nm) irradiation. Again, the degradation performance of pure SnO_2_ is very low due to its high-band-gap nature. The composite materials showed much improved photoactivities, and the optimized 6SnO_2_/g-C_3_N_4_ sample showed 42% degradation activities under the given conditions as shown in Figure 3B. We also carried out the stability test of the optimized sample for five consecutive cycles, each cycle composed of a 5-h run. It is obvious from Figure 3C that there is no detectable decrease in the H_2_ production activities, indicating that the optimized sample is very stable.

### Charge Separation

**Figure 4:**
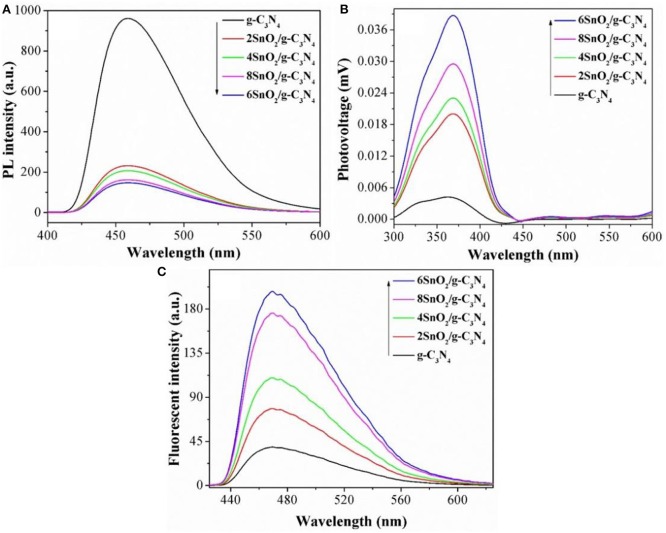
Photoluminescence spectra **(A)**, SS-SPS **(B)**, and coumarin fluorescent spectra **(C)** of pure g-C_3_N_4_ and XSnO_2_/g-C_3_N_4_.

The improved photoactivities of the composites compared to pure g-C_3_N_4_ are attributed to the extended charge separation. In order to determine it, we carried out PL spectra, keeping excitation λ at 390 nm. It is clear from Figure 4A that g-C_3_N_4_ gives intense peak at 470 nm, indicating poor charge separation. However, the intensities of the composites are progressively decreased as the amount of SnO_2_ nanoparticles is increased, and the optimized sample shows relatively low PL peak, indicating excellent charge separation in it (Zhang et al., 2015; Ali et al., 2016; Lu et al., 2018; Ali N. et al., 2019; Ali S. et al., 2019). The relatively low intensities of PL peaks indicate enhanced charge separation and are responsible for improved photoactivities.

We further extended the charge separation experiments by measuring the atmosphere-controlled steady state surface photovoltage spectra (SS-SPS), and the results are shown in Figure 4B. As evident, g-C_3_N_4_ shows very low SPS intensity. However, the SPS peak intensities are much improved when both g-C_3_N_4_ and SnO_2_ are coupled and the optimized 6SnO_2_/g-C_3_N_4_ sample shows the highest peak intensity. Since high is the intensity of the SPS peak, high is the charge separation (Zada et al., 2018, 2019a,b); therefore, we can say that the composites impart enhanced charge separation and contributing to the improved photoactivities.

We also measured the hydroxyl radical (·OH) activities of the fabricated samples by doing coumarin fluorescent experiments under visible-light irradiation. Since ·OH is the major contributor to charge separation during photocatalysis and react with coumarin to form 7-hydroxycoumarin; therefore, the higher the intensity of coumarin fluorescent peak, the higher is the charge separation. As can be seen from Figure 4C, pure g-C_3_N_4_ gives very low peak, which shows its inherited low charge separation. However, the ·OH radical activities are considerably improved when both g-C_3_N_4_ and SnO_2_ are coupled, indicating improved charge separation and hence extended photoactivities (Ali et al., 2018a; Yasmeen et al., 2019a).

## Discussion

**Figure 5:**
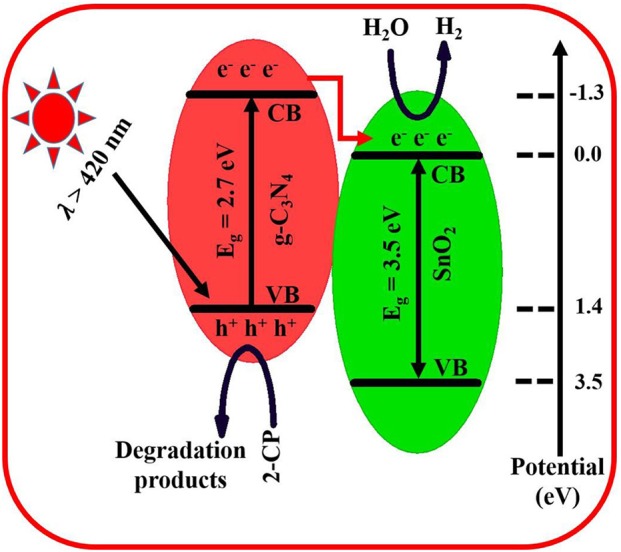
Schematic representation of charge separation, H_2_ production, and pollutant degradation by 6SnO_2_/g-C_3_N_4_.

The improved charge separation in the prepared composite results in the enhanced H_2_ production and 2-CP degradation. This enhanced charge separation has been schematically shown in Figure 5. The band gap of g-C_3_N_4_ is about 2.7 eV and absorbs visible-light photons (Raziq et al., 2015, 2017). Its CB present at −1.3 eV is most suitable for H_2_ production and superoxide generation which require reduction potential of 0.00 and −0.33 eV, respectively. Its VB is present at 1.4 eV (Yasmeen et al., 2019b). On the other side, the band gap of SnO_2_ is 3.5 eV, and its CB is present below the CB of g-C_3_N_4_ (Zada et al., 2016). Under visible-light irradiation, only g-C_3_N_4_ is excited, and electrons jumped to its CB, leaving positive holes in the VB. Since the excited electrons have a very short lifetime; therefore, they jumped to the CB of SnO_2_ to achieve some stability for a while. Here these electrons reduce water into H_2_ while the holes in VB of g-C_3_N_4_ are solely left to carryout oxidation of alcohol. In case of 2-CP degradation, these positive holes either directly oxidize pollutants or undergo the formation of more reactive species such as hydroxyl-free radicals, which then finally degrade the target pollutant into simple CO_2_ and water (Zada et al., 2018).

## Conclusion

In order to overcome energy crises and environmental pollution, here, we synthesized g-C_3_N_4_ nanosheets and coupled them with SnO_2_ nanoparticles. The optimized composite of 6SnO_2_/g-C_3_N_4_ produced about 132 μmol of H_2_ from water in 1 h and degraded 42% 2-CP pollutant under visible-light irradiation as compared to the photoactivities of bare g-C_3_N_4_ and SnO_2_. These enhanced photoactivities are attributed to the better charge separation as the excited electrons thermodynamically transfer from g-C_3_N_4_ to SnO_2_ as has been confirmed from photoluminescence spectra, steady state surface photovoltage spectroscopic measurement, and formed hydroxyl radicals. It is believed that this work would provide a feasible route to synthesize photocatalysts for improved energy production and environmental purification.

## Data Availability Statement

The raw data supporting the conclusions of this article will be made available by the authors, without undue reservation, to any qualified researcher.

## Author Contributions

All authors listed have made a substantial, direct and intellectual contribution to the work, and approved it for publication.

### Conflict of Interest

The authors declare that the research was conducted in the absence of any commercial or financial relationships that could be construed as a potential conflict of interest.
